# Establishment, operation and development of the electronic Pollen Information Network (ePIN) in Bavaria, Germany

**DOI:** 10.1038/s41598-025-90275-x

**Published:** 2025-04-10

**Authors:** Susanne Kutzora, Antje Strasser, Katharina Grenzebach, Ramona Gigl, Robert Gebauer, Alisa Weinberger, Julia Szperalski, Caroline Herr, Caroline Quartucci, Stefanie Heinze

**Affiliations:** 1 Department for Occupational and Environmental Medicine, Bavarian Health and Food Safety Authority, Pfarrstr. 3, 80538 Munich, Germany; 2 Institute and Clinic for Occupational, Social and Environmental Medicine, University Hospital, LMU Munich, Ziemssenstr. 1, 80336 Munich, Germany; 3 IT Consulting Robert Gebauer, Munich, Germany

**Keywords:** Electronic pollen monitoring, Biomonitoring network, Pollen, Allergy, Climate change, Health services, Epidemiology

## Abstract

Current data of actual airborne pollen concentrations is of high value for allergy sufferers and medical practitioners, as allergen avoidance is an important therapeutic approach to allergic rhinitis. In addition, pollen concentrations are important indicators for monitoring climate change, providing information on changes in plant growth, plant reactions to climate change and the arrival of neophytes. The importance of pollen monitoring as well as technical developments and the emergence of electronic pollen monitors led to the establishment of the electronic Pollen Information Network (ePIN) in Bavaria, Germany. ePIN is a fully electronic pollen monitoring network that offers real-time pollen information to the public currently in three-hourly intervals. It consists of eight electronic pollen monitors as well as four Hirst-type pollen traps. The ePIN data is public and free of charge. Consumers can access the information via a website or an app. This article describes ePIN, its establishment, operation and further development.

## Introduction

In Germany about 12 million adults suffer from allergic rhinitis and the number is increasing^1^. More than one million children and adolescents are affected by allergic rhinitis^2,3^. 37% of all adolescents in Germany are sensitized to inhalant allergens such as pollen from timothy grass, rye, birch or mugwort^3^. Sensitization in adolescents was found to be persistent in a 10-year observation and sensitization rates did not decrease in adults^1^. Allergies can result in worse quality of life, asthma and high medical costs as well as indirect costs associated with lost work productivity^4^. Due to the high prevalence and resulting negative consequences, airborne allergies are a very important public health issue and adequate measures for preventive and therapeutic approaches are needed. Allergen avoidance is an important causal therapy procedure and patients need an individual allergy management. Therefore knowledge about the patients actual current allergen exposure is required^5^.

In Germany, pollen data has mostly been collected by Hirst-type pollen traps^6,7^. Hirst-type pollen traps are recognized as the current standard for pollen monitoring delivering volumetric information of pollen per m^3,8,9^. When working with Hirst-type pollen traps, pollen must be analyzed manually, which takes three to nine days before results can be presented, and manual pollen counting can lead to qualitative differences^10,11^. In most cases, the locations of the pollen traps are not planned as an area covering network. Allergy sufferers therefore have unreliable data on pollen concentrations at their actual location and time^12^.

In recent years, there have been many innovations in the field of pollen measurement technologies^13–15^. Electronic pollen monitors were developed, that showed a positive correlation coefficient (r = 0.98; p < 0.01) between their reported daily pollen concentrations and the pollen concentrations reported by Hirst-type pollen traps^16^. The electronic pollen monitors can provide near real-time information. In 2015, the Bavarian State Government commissioned the Center of Allergy and Environment (ZAUM) at the Technical University of Munich to develop a scientifically based pilot network for a statewide pollen monitoring. In a preliminary study, ZAUM recommended the electronic pollen monitor type BAA500 for the use within the network as result of market exploration and comparison with Hirst-type pollen traps^16^. The best possible locations for the electronic pollen monitors were determined by a cluster analysis of a dense network with 27 Hirst-type pollen traps according to the pollen distribution in Bavaria and the distribution of the population^7^.

An accordingly distributed statewide network of electronic pollen monitors on a long-term basis can provide locally representative, up-to-date pollen information for the public and research purposes, and can improve pollen forecasts and data for health and climate research. In 2016, the Bavarian Health and Food Safety Authority (Bayerisches Landesamt für Gesundheit und Lebensmittelsicherheit, LGL) was assigned with the establishment and supervision of a permanently installed electronic pollen information network (ePIN) in Bavaria.

However, there are similar real-time pollen monitoring initiatives in Europe where data is shared with the public. For example, there are real-time data pollen measurements in Croatia, in Switzerland, in Lithuania, in Great Britain, in Finland and in other countries. There are also measurements of pollen counts on all continents worldwide^12^.

This paper aims to describe in detail the establishment, operation and ongoing development of the electronic Pollen Information Network (ePIN) in Bavaria.

## ePIN – establishment and data

The LGL had implemented ePIN since 2016 and started its operation in 2019.

### Locations

**Fig. 1:**
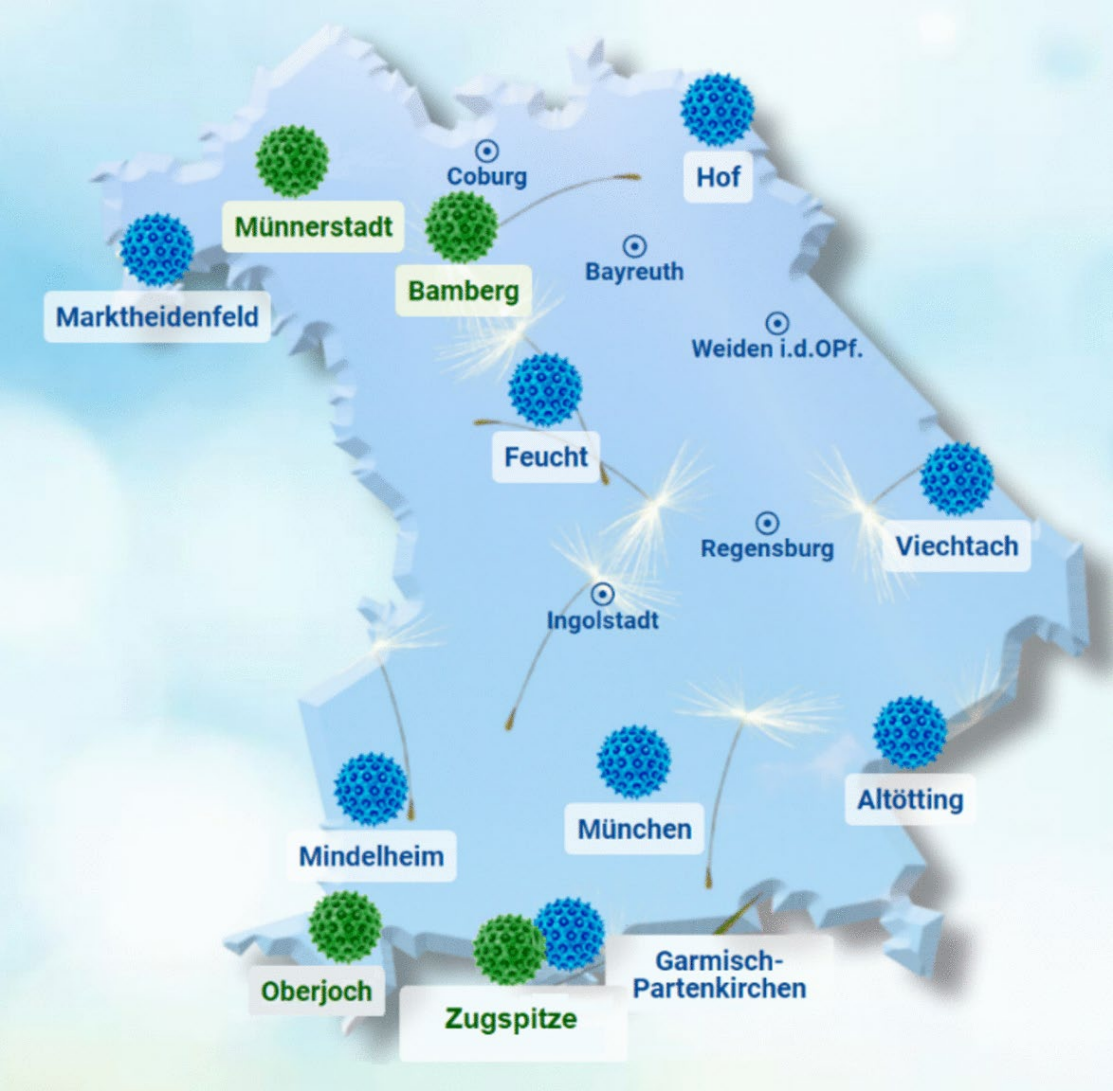
Eight electronic pollen monitors (blue), four manual Hirst-type pollen traps (green) The map was generated with the ePIN Website, Version 4.0.3 (https://epin.lgl.bayern.de).

ePIN consists of eight electronic pollen monitors as well as four Hirst-type pollen traps (see Fig. 1).

The allocation of the electronic pollen monitors in Bavaria was the result of a preliminary study^7^. Technical and logistic requirements had to be met for the installation of the pollen monitors. Standard conditions for Hirst-type pollen traps are defined in a European consensus paper (VDI guideline, DIN CEN/TS 16,868, March 2016). Based on these standards, LGL has defined additional criteria necessary for the sites of electronic pollen monitors (Table 1).

**Table 1:** Catalog of criteria for inclusion of electronic pollen monitoring sites in ePIN based on VDI guideline, DIN CEN/TS 16,868, March 2016.

Requirements according…		
…Site	…Location	…Emission sources
Flat horizontal surface	Secured and fall-proof access ways and working areas at the pollen monitor	No overrepresentation of one type of pollen in its surrounding
Higher than other surrounding roofs or wind obstacles	Moisture protected electricity connection (household electricity sufficient, emergency power supply advantageous, but not necessary)	Absence of highly anemophile sources: uncut areas of grass (> 50 m) and birch (> 100 m)
Between 9 to 15 m above ground level (for example on a roof)	Internet access via LAN or powerful cellular transmission (high volume of data!)	Absence of high non-biological sources of particle emissions in its surrounding
> 2 m distance to the edge of the building	Permanent preservation of conditions	Absence of wind turbulence sources in its surrounding
More than 150 cm over the ground of the roof/ elevated surface	Lightening protection	Yearly status analysis of land use and evaluation if requirements are still met
	Structural analysis for loading capacity including safety margin	

According to the defined criteria in Table 1, possible sites were screened with the Light Detection and Ranging LIDAR data from the Bavarian Surveying and Mapping Authority. Most of the buildings identified as ePIN sites were public buildings like hospitals or public schools.

Further the LGL and ZAUM validated ePIN in 2018^17^. In addition to the eight electronic pollen monitors, eight Hirst-type manual pollen traps were installed at the same locations and run for one pollen season in 2018. After the results had been set, the eight manual pollen traps were removed.

Regardless of the eight manual pollen traps that had been run during this validation period, four Hirst-type pollen traps are permanently operated at locations where the German Pollen Information Service foundation (Stiftung Deutscher Polleninformationsdienst, PID) has been carrying out pollen count measurements for decades. The continued operation of these four Hirst-type pollen traps is important to continue the data series needed to monitor climate change, to identify emerging pollen from invasive plants and to monitor the long-distance transport of pollen^18^.

The Hirst-type pollen traps are located in Bamberg, Münnerstadt, Oberjoch and the Environmental Research Station *Schneefernerhaus* just below the summit of Zugspitze. Bamberg started pollen monitoring in 1989, Münnerstadt in 1991, Oberjoch in 1995 and *Schneefernerhaus* in 2015. The *Schneefernerhaus* is the best observation point for high-altitude flying pollen and long-distance transport at 2652 m^19,20^. Specific conditions such as lower temperatures and lower air pressure made it necessary to equip the Hirst-type pollen trap with an additional heater and to replace the suction pump with a model suitable for alpine use^21^.

### Pollen measured in ePIN

Pollen with allergenic potential, that had not been important allergenic pollen for allergy sufferers in a certain region, can become important allergy triggers when pollen concentrations or allergen pollen potency change. Therefore, ePIN measures the most important allergenic pollen for people in Bavaria, pollen with future allergenic potential and pollen that could be relevant for the monitoring of climate change (Table 2). Pollen detection takes place at different taxa levels due to the constant development of the software. Some pollen can already be assigned to the associated genus and others are classified at family level.

**Table 2:** Pollen measured in ePIN (printed in bold: Main allergenic pollen in Bavaria).

Pollen and spore measured in ePIN			
**1**	**Hazel (*Corylus*)**	21	Cleavers (*Galium*)
**2**	**Alder (*Alnus*)**	22	Hop (*Humulus*)
**3**	**Grasses (*Poaceae*)**	23	Balsam (*Impatiens*)
**4**	**Birch (*Betula*)**	24	Walnut (*Juglans*)
**5**	**Rye (*Secale*)**	25	Larch (*Larix*)
**6**	**Mugwort (*Artemisia*)**	26	Spruce (*Picea*)
**7**	**Ragweed (*Ambrosia*)**	27	Pine Family (*Pinaceae*)
**8**	**Ash (*Fraxinus*)**	28	Pine (*Pinus*)
9	Fir (*Abies*)	29	Plantain *(Plantago*)
10	Maple (*Acer*)	30	Plane (*Platanus*)
11	Horse Chestnut (*Aesculus*)	31	Cottonwood (*Populus*)
12	Composite Plants (*Asteraceae*)	32	Oak (*Quercus*)
13	Crucifer (*Brassicaceae)*	33	Holm (*Quercus ilex*)
14	Chestnut (*Castaneae*)	34	Sorrel (*Rumex*)
15	Goosefoot (*Chenopodium*)	35	Willow *(Salix*)
16	Hornbeam (*Carpinus*)	36	Elder (*Sambucus*)
17	Sedge Family (*Cyperaceae*)	37	Yew (*Taxus*)
18	Heather (*Erica*)	38	Limetree *(Tilia*)
19	Beech (*Fagus*)	39	Elm *(Ulmus*)
20	Fungi (*Fungus*)	40	Nettle (*Urtica*)

### Practical steps building ePIN

Each selected site (see 2.1. Locations) was visited, inspected, and modified to meet requirements shown in Table 1. The installation of the electronic pollen monitors included the following tasks:

- Leasing and maintenance contracts for eight electronic pollen monitors type BAA500 with manufacturer
- Tenancy agreements with the according building management for the approximately 1.5 m^2^ roof areas. Bigger areas can be necessary in case of the installation of a podium or other set ups for occupational safety
- Structural analysis of every roof with platforms made of steel for load transfer checks
- Building the foundations for the pollen monitors on the roofs
- Building secured working conditions by installing handrails, walkways, barriers
- Transporting the monitors (355 kg) onto the roofs via crane
- Installing lightning protection
- Ensuring electronic power on the roof
- Ensuring internet access for data transfer and remote maintenance
- Building a data infrastructure (see 2.4. Data)
- For each of the 8 locations, several contracts needed to be established:
  - A leasing contract with the manufacturer of the pollen monitors
  - A rental agreement for the storage unit on the building’s rooftop,
  - A contract with the internet service provider,
  - An agreement with the individual responsible for changing magazines on-site and minor actions on the pollen monitor like a restart (typically an employee of the respective health authority on the roof of which the pollen monitor had been installed).

Some special considerations must be taken into account when carrying out practical installations. For instance, power lines should be installed with provisions for independent billing. Pollen monitors must be connected to the internet, and in older buildings, heritage protection regulations must be respected. In hospitals, it is essential to keep emergency exits accessible at all times, adhere to strict hygiene requirements, and prioritize patient safety. Consequently, construction work should be scheduled on weekends and be ready to halt immediately in case of emergency. Additionally, schedules may need to be adjusted for adverse weather conditions, such as snow, ice, rain, or wind.

When installing the monitors in ePIN, a safety and health coordinator ensured that occupational health and safety regulations were followed during the construction.

**Fig. 2:**
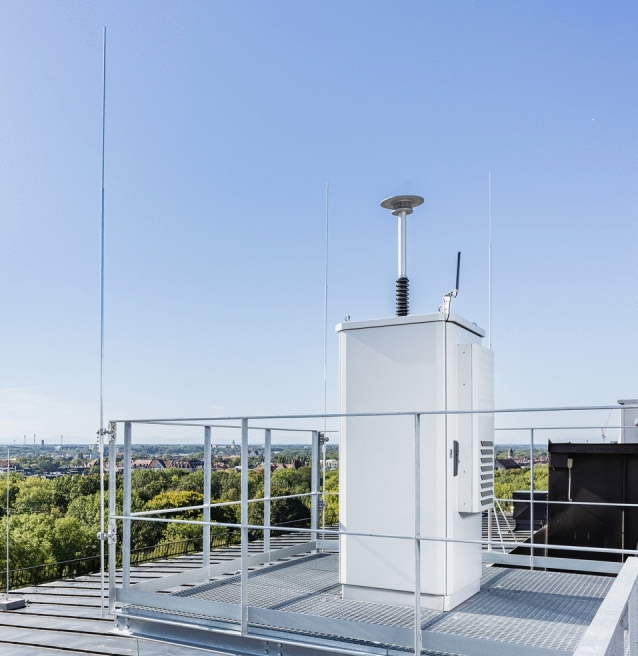
Pollen monitor with required installations, © LGL, Alexander Harand.

An installed operable pollen monitoring station is shown in Fig. 2.

At each site one responsible contact person was commissioned, briefed and given the tasks of changing the sample carrier repository on time, executing manual restarts if necessary, and delivering the sample carriers. Most contact persons were acquired from local health departments.

At the sites with Hirst-type pollen traps, the sample change needs to be carried out manually every week, as well as the air intake has to be cleaned and the set up needs to be controlled. Each station attendant was briefed and assigned with the weekly maintenance.

The electronic pollen monitors are maintained remotely by the manufacturer as well as during an annual manual inspection.

If a public authority operates such a pollen monitoring network, it should, like the LGL, be responsible for the functioning of the network with its’ pollen monitors, IT infrastructure, concept and maintenance of website and app, promotion, project management and research collaborations. LGL also monitors disturbances, coordinates checkups, informs and trains the support staff, regularly reviews sites and requirements and initiates adjustments or changes as necessary, and steps in during emergencies.

### Data

**Fig. 3:**
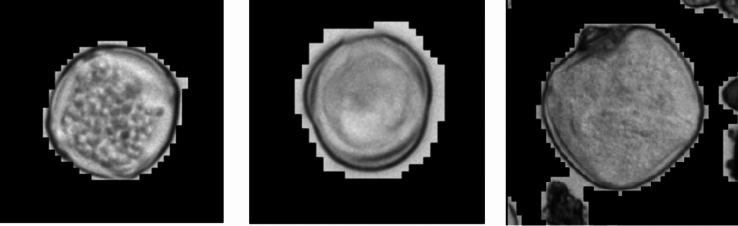
Synthesized pictures of Betula, Artemisia and Fagus pollen. The data from the manual Hirst-type pollen traps must be entered into the database manually.

The data infrastructure is located at the Leibniz Supercomputing Centre of the Bavarian Academy of Sciences and Humanities (LRZ). Each electronic pollen monitor is connected to the Internet and data is fully automatically transmitted and processed, stored and archived at the LRZ. Each picture of each detected particle is saved as well as the pollen concentration (pollen/m^3^/timestamp) and can be used for later analysis. The transferred data volume per electronic pollen monitor depends on the numbers of pollen counts and can easily reach up to 50 gigabytes per day and device during high season. Buffer zones are built in for the event of high data load, so that data processing queues can be resolved without manual intervention. The electronic pollen monitor with mobile data contract has an external hard drive with five terabytes that is replaced every three months, while pollen concentration data is transferred in real-time using the mobile connection. The picture stacks used for pollen identification are locally saved and later uploaded to the LRZ via direct internet access later on. The picture stacks are several different images of one pollen, that are used to synthesize one pollen picture that enables to identify the pollen (Fig. 3). To save data volume, the hard drive is replaced every three months together with the sample carrier repository.

All ePIN data is publicly available via a standard interface (REST-API) described at https://epin.lgl.bayern.de/schnittstelle. The ePIN REST Interface allows querying locations and pollen concentrations from ePIN. The interface offers three endpoints. Each endpoint can be called with the HTTP method “GET”. The response is returned with mime-type application/json. Time intervals in the request and in response are specified as POSIX time from start to finish. Data are available from May 2019 of all eight electronic pollen monitors and for the Hirst-type pollen traps from over 20 years. In Bamberg there are several gaps in the data. Pollen images are also expected to be available via a REST- API in 2024.

**Fig. 4:**
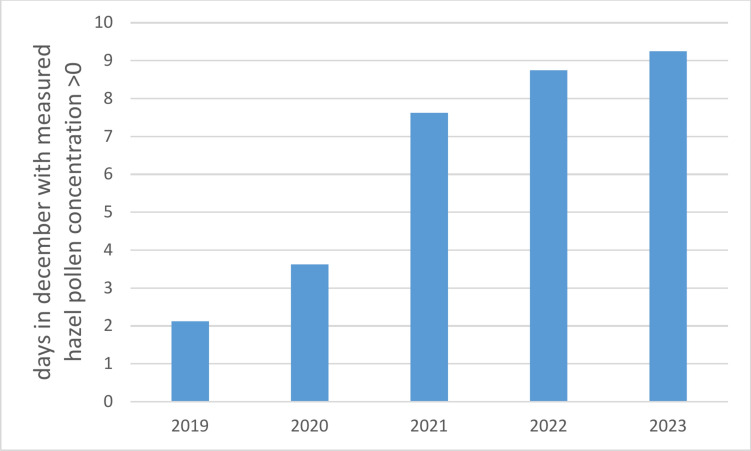
Days with measured hazel pollen concentration > 0. Mean values across all eight electronic pollen monitors in December 2019–2023.

The monitoring takes place during the whole year with data available for each date, so that possible changes of pollen seasons can be detected. Until 2022, pollen samples were reduced to one sample per day in winter period from November to mid- February. Due to climate change and the associated warmer temperatures in the winter months, the pollen season is likely to start earlier. Accordingly, an increase in the number of days with measured hazel pollen concentrations in December was observed in ePIN (Fig. 4). The temperature increase due to climate change and the resulting earlier blooming of hazel could be responsible for this observation. The increase in measured pollen concentration could also possibly be partly attributed to factors such as advances in detection software. However, this trend has also been documented in other studies^22–24^.

The eight electronic pollen monitors recorded hazel pollen on an average of 2.125 days in December 2019, increasing to an average of 9.25 days in December 2023. This indicates an increase in the number of December days with measurable hazel pollen over these years (Fig. 4).

**Fig. 5:**
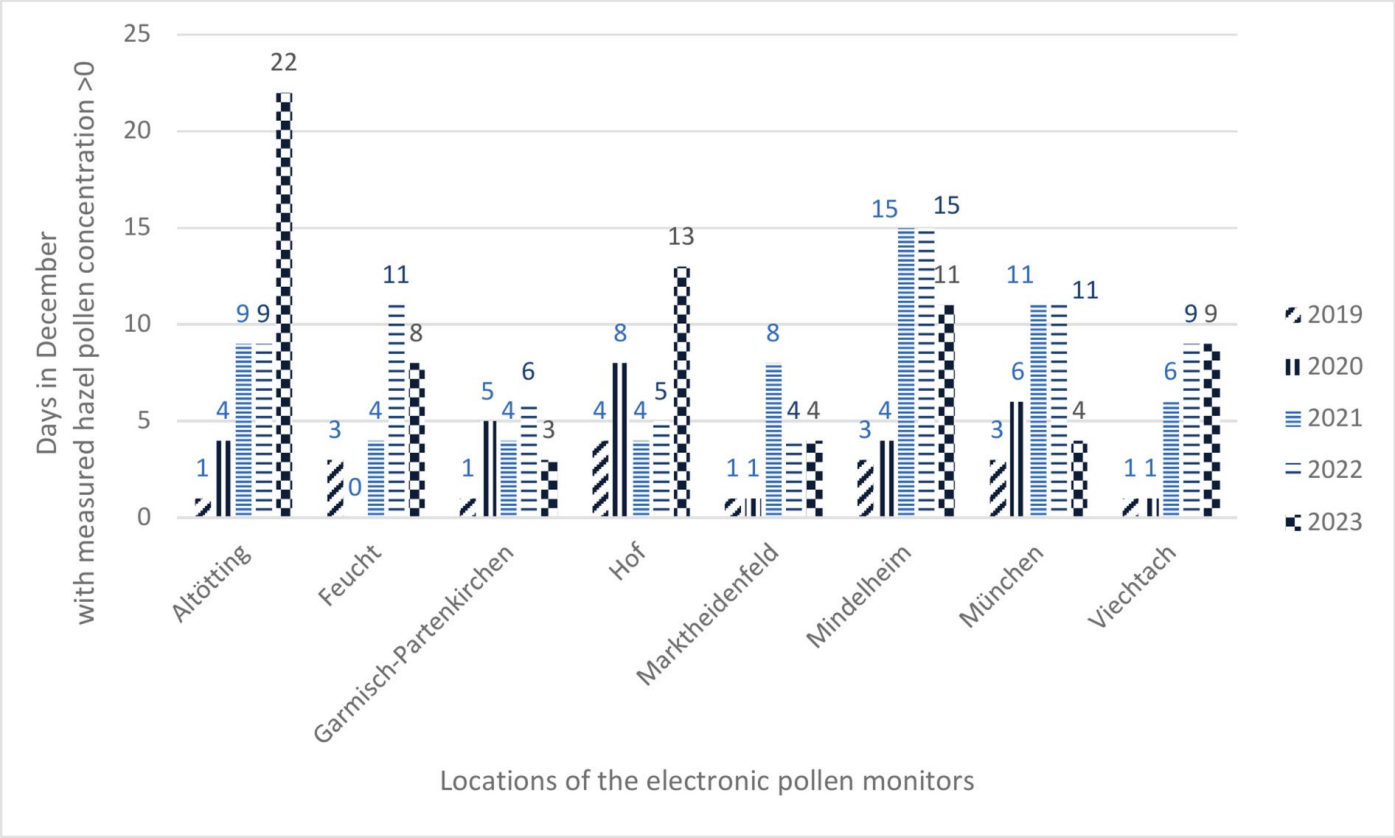
Number of days with measured hazel pollen concentration > 0 at all eight sites in December 2019–2023.

Regional differences due to the differing conditions in the various pollen flight zones show the importance of monitoring pollen at the eight sights (see Fig. 5). For example, in Mindelheim the concentration of hazel pollen in general appears to be higher than in Garmisch-Partenkirchen. In addition, Fig. 5 shows that the number of days with hazel pollen count increased at essentially all locations between 2019 and 2023.

### Website and app

In May 2019, ePIN was officially launched together with the ePIN-website (available via www.epin.bayern.de). The release of the ePIN-app followed in 2020. The app was launched with the aim to provide high quality data to interested individuals without having to visit the website every time. The app is based on the same source code as the website. User feedback at the app stores is continuously evaluated and used for improvements.

We deliberately refrain from specifying threshold values that might indicate a correlation between pollen concentrations and allergic symptoms. Current scientific findings on the precise pollen concentration that triggers allergic reactions remain heterogeneous, as allergic responses vary individually. Individuals may exhibit different reactions to pollen exposure depending on their unique physiological state and varying temporal circumstances. The concentration of pollen necessary to elicit a response can fluctuate, and the allergenic potential of individual pollen grains—even within the same species—may differ. Additionally, symptoms may present with a delayed onset, potentially correlating with pollen levels from preceding days.

As part of a systematic literature review, the LGL evaluated findings from 31 studies^25,26^. The review focused on the association between pollen concentrations of hazel, alder, ash, birch, grasses, mugwort, and ragweed and related health outcomes. These outcomes were defined as self-reported symptoms, increased medication usage, more frequent doctor visits, or higher emergency admission rates. For ash, birch, and grass pollen, evidence was found indicating correlations between specific pollen concentrations and adverse health outcomes. However, the analysis revealed that the validity of threshold values as a measure is limited due to variable study quality. No conclusive threshold values were identified for hazel, alder, mugwort, and ragweed pollen.

Given the current evidence base, further research is necessary to comprehensively assess the relationship between measured pollen concentrations and their associated health impacts. From the LGL’s perspective, defining symptom-relevant threshold values for pollen concentrations is not currently feasible. Continuous investigation is essential for better correlating pollen levels with health outcomes.

**Fig. 6:**
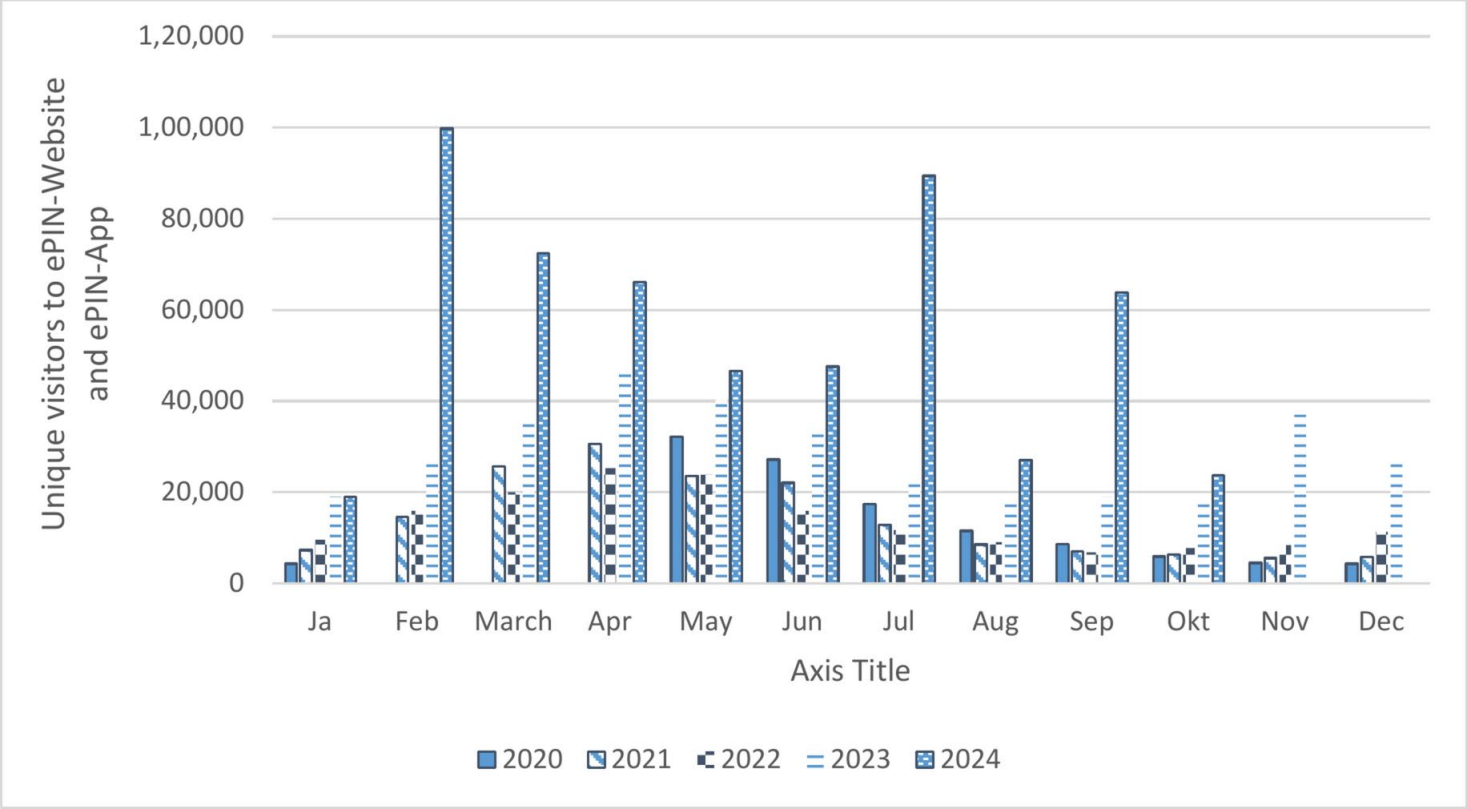
Unique visitor numbers of the ePIN Website and app from May 2019 until October 24 (missing data in 2020 because of server alterations).

Figure 6 shows the number of unique visitors (IP address is counted once during reporting period) to the ePIN website and app since the launch in May 2019. The numbers are distributed according to the pollen season, starting with low numbers in January, rising from March with a peak in July, slowly decreasing until September, and ending with low numbers from October until December. After the first full year of data, fewer visitors were counted in 2020 than in 2019. After app updates and news in the media, visitor numbers in 2023 show the highest increase compared to the other years.

Regarding data privacy the pollen concentration data is freely available and user data is only collected in the form of the IP address. IT security measures are in accordance with the responsible official IT authority.

### APOLLO study

Studies showed the importance of an individualized approach to the interpretation of pollen concentrations for pollen allergy sufferers (Becker et al., 2021; Steckling-Muschack et al., 2021). To support the self-management of patients with pollen allergies, an individualized app was developed, which was part of the so-called *APOLLO study* for health monitoring of pollen allergy sufferers^27^.

The aim of the *APOLLO study* was to complement the ePIN app with helpful individualized content concerning pollen allergy sufferers. The APOLLO app includes a calendar overview and a questionnaire for a daily entry into a logbook of allergy-related symptoms and complaints. In the app, two indices are created based on the data from the logbook. One index shows the subjective assessment of the severity of the physical symptoms (itchy nose, watery eyes, etc.); the other index shows the severity of the complaints in everyday life (impairment in sleep or concentration, etc.). The indices are graphically processed and show the individual symptoms next to the pollen concentrations of ePIN up to that point. Before using the app, the research participants had to complete a survey about their health status, medication, quality of life, use of pollen information services, and life circumstances. Depending on their responses about their allergies, participants were assigned a specific time slot of 6 weeks in which to use the APOLLO-app and complete the logbook. At the end of the 6 weeks, participants completed a second questionnaire regarding health competence, their feedback on ePIN, and usability of the APOLLO app^27^.

A symptom diary based on the APOLLO app has been integrated into the ePIN products (website and app).

### Possible expansion of the ePIN network (*ePIN-Plus*)

*ePIN-Plus* offers private stakeholders the opportunity to join the ePIN network by setting up their own electronic pollen monitor. Private monitors can supply their data to the ePIN network as long as the site, electronic monitor, and data meet the standards of the network. An agreement may be entered into that includes the criteria defined in Table 1. Furthermore, the use of the electronic pollen monitor must be granted for at least four years, and all data must be transferred to the ePIN server without alterations whilst failure-free operation as far as possible.

## Further activities associated with ePIN

### Data modelling (*ePIN-Nowcasting*)

In order to be able to calculate pollen concentrations at locations that lie between two or more electronic pollen monitors, an interpolation model is being tested by ZAUM, assigned by LGL, in the project *ePIN-Nowcasting*. Data from a previous study in Günzburg, Germany, were used to validate the model. Daily pollen concentration was modeled for locations where there was no pollen monitor within 30 km using the variables pollen concentration, altitude and rainfall^28^.

### Pollen forecast (*ePIN-Opt*)

The aim of the project *ePIN-Opt* is to develop pollen count forecasts based on ePIN data. The project is being implemented by ZAUM with the project partner LGL since 2022. Up to this point, pollen forecasts in Germany have been based on retrospective pollen concentrations from the German Pollen Information Service Foundation—Network, phenological observations, and short- and medium-term weather forecasts from the German Weather Service (Deutscher Wetterdienst, DWD). The System for Integrated Modeling of Atmospheric Composition (SILAM)^29^, that was developed for a European forecast of pollen flight by the Finish Meteorological Institute (https://silam.fmi.fi/pollen.html) is used and complemented by ePIN data as well as high resolution vegetation-maps necessary for an accurate forecast^30^. ePIN data extends the data base on which SILAM is built, as the data is available in real time. Calibration of the forecast can be carried out using ePIN data.

The *ePIN-Opt* project has an additional objective: ensuring the comparability of archived data with data generated by the improved software used in pollen count analysis (see 3.4 Artificial intelligence (AI) based software). Due to enhancements in the software, direct comparisons with historical datasets are currently not feasible. It is therefore planned that a method will be developed to enable all ePIN data to be analyzed with the software version currently in use. This will allow for the re-evaluation of all archived datasets using the updated software. This method can be applied each time the software is upgraded, ensuring that data records remain comparable over time.

### AutoPollen programme

The European Meteorological Services Network (EUMETNET) launched the *AutoPollen programme* in 2018^31^. The programme aims to develop an automatic European pollen and fungal spore monitoring network by integrating ongoing projects and defining the main standards of automatic pollen and fungal monitoring. The LGL participates in the *AutoPollen programme*. Since the establishment of ePIN several new automatic pollen monitors have been developed. From March to July 2021 an *Intercomparison Campaign* took place in Munich as part of the *AutoPollen programme*. Nine types of electronic monitors as well as four Hirst-type pollen traps were compared at the same site. The BAA500 and Swisens Polenos performed best in classifying individual pollen taxa of *Betula*, *Fraxinus*, Poaceae, and *Quercus*, with the smallest differences (p <  = 0.001) from the Hirst-type pollen trap results^32^. It is planned to repeat the comparative studies for continuous evaluation.

### Artificial intelligence (AI) based software

The manufacturer Helmut Hund GmbH regularly updates the software of the BAA500. Since 2021, a software based on artificial intelligence has been run in addition to the previous software on the electronic pollen monitors. The analyses based on the AI software were part of the *Intercomparison Campaign* by the European *AutoPollen programme*. The data analyzed by the AI software showed better agreement with the data of the Hirst-type pollen traps than the preceding software^32^. In ePIN the new software has been in use since 31 October 2024.

## Discussion

The importance of pollen monitoring, as well as technological development led to the establishment of the electronic pollen information network (ePIN) in Bavaria, Germany. ePIN is one of the first fully electronic pollen monitoring networks worldwide that offers pollen information to the public currently in three-hourly intervals. The establishment of ePIN provides various strengths. In order to keep the network running, a well-functioning infrastructure has been built that can also be used in the future for further developments. Through continuous and reliable measurement of pollen by public authorities, climate change monitoring can be further advanced. The pollen data in ePIN is freely accessible for everyone including scientists and practitioners and therefore can serve as a database for research. ePIN’s rising user numbers for the app and website as well as positive user feedback indicate a high demand for pollen count information.

ePIN provides valuable data near real time in Bavaria giving allergy sufferers the opportunity to plan their daily life according to pollen concentrations. Compared to manual Hirst-type pollen traps with long time lags between pollen collection and the existence of results of the pollen analysis, ePIN therefore has a major advantage^33^.

However, there are limitations in ePIN and the network has potential to be further developed. A group on Earth Observations (GEO) focusing on end user requirements stated that pollen counting “need to be improved, aiming at more accurate and advanced forecasting”^34^.

The eight electronic pollen monitors of ePIN cover the pollen flight in Bavaria in a representative way. Nevertheless, if a person is between two or more monitors, it is currently not unambiguous which data to use. To address this problem data modeling is being developed in the project *ePIN-Nowcasting*. Hirst-type pollen traps are the standard procedure of measuring pollen concentrations and are therefore used for validation. However, a real objective validation method evaluating the pollen counts by the electronic pollen monitors and the real amount of pollen in the air has not yet been developed and could be a task for the future^16^.

Besides the establishment of ePIN using the technology of image recognition, further electronic networks using laser technology have been implemented, for example in Japan^35^, and in Switzerland^36^. All electronic monitors use the technique of image recognition and/or air-flow cytometry^33^. Due to the design of the pollen monitors using air-flow cytometry, further tests of the trapped particles can be more easily added than with monitors using image recognition. The test-tube can easily be expanded and further tests can be added. Image recognition is a validated method detecting pollen. If other research questions regarding the study of particles in the air are of interest, the laser using techniques might be favorable.

Electronic pollen monitoring networks have to be seen as a long-term investment with further expenditures. As technology is innovative, it is necessary to keep up with developments in order to maintain the state of the art. ePIN therefore takes part in the *AutoPollen programme*^31^ and participates in cooperative studies like the *Intercomparison Campaign*^32^.

We acknowledge the limitations of AI-based pollen monitoring systems, particularly the challenges in accurate pollen identification and the need for continuous training and validation of the algorithms. As a public health authority, we rely on the data provided by the manufacturer and have no influence on the development or improvement of the underlying AI technology. Ensuring ongoing quality assessment and addressing these limitations remains crucial and should be prioritized by device manufacturers and AI developers.

Another challenge is the appropriate risk communication, so that end users benefit from the electronic network. Risk communication should be proactive, trustworthy, complete, transparent and understandable for the target groups^37^. In the future, we aim to reach more allergy sufferers and practitioners. Assuming the same prevalence of allergic rhinitis in Bavaria as in Germany, about 1.2 million adults in Bavaria suffer from allergic rhinitis. By adding an individual symptom diary to the ePIN-app and website, user numbers could be increased and help more allergy sufferers manage their allergy.

## Conclusion

ePIN is one of the world’s first electronic pollen monitoring networks offering its data to the public and for scientific research purposes The aim is to improve pollen count information for allergy sufferers in Bavaria. ePIN offers eight electronic pollen monitors at scientifically selected sites, offering data for different locations in Bavaria and current pollen data for up to three hours. ePIN has been under continuous development for over eight years, during which substantial expertise has been accumulated. The selection process for optimal locations and site-specific requirements has been refined, overcoming numerous practical challenges along the way. The development remains ongoing, with LGL striving to enhance ePIN’s services for allergy sufferers. Efforts include refining the app’s user experience and creating a personalized symptom diary that seamlessly integrates with comprehensive pollen data. Pollen monitors are continuously improved through developments in software and hardware. There are many aspirations to build electronic pollen monitoring networks. The work at LGL aims to run ePIN on the current state of the art in research and technology, as well as to develop its services. Collaboration and exchange are important to the quality of the network. We hope that the information in this article will support others in building their networks.

## Data Availability

The data that support the findings of this paper are openly available at https://epin.lgl.bayern.de/schnittstelle.

## Abbreviations

DWD: German Weather Service (Deutscher Wetterdienst)
ePIN: Electronic Pollen Information Network (Elektronisches Polleninformationsnetzwerk Bayern)
EUMETNET: European Meteorological Services Network
LGL: Bavarian Health and Food Safety Authority (Bayerisches Landesamt für Gesundheit und Lebensmittelsicherheit)
LRZ: Leibniz Supercomputing Centre of the Bavarian Academy of Sciences and Humanities (Leibniz-Rechenzentrum)
PID: German Pollen Information Service Foundation (Stiftung Deutscher Polleninformationsdienst, PID)
SILAM: System for Integrated Modeling of Atmospheric Composition
ZAUM: Center of Allergy and Environment (Zentrum Allergie und Umwelt, Technische Universität und Helmholtz Zentrum München)
